# Time me by the moon: The evolution and function of lunar timing systems

**DOI:** 10.1038/s44319-024-00196-5

**Published:** 2024-07-16

**Authors:** Andrés Ritter, Kristin Tessmar-Raible

**Affiliations:** 1 Laboratory of Integrative Biology of Marine Models, UMR8227 Sorbonne Université-CNRS, Station Biologique de Roscoff, 29688 Roscoff, CEDEX France; 2 Max Perutz Labs, University of Vienna, Vienna BioCenter, Dr. Bohr-Gasse 9/4, 1030 Vienna, Austria; 3 Alfred Wegener Institute Helmholtz Centre for Polar and Marine Research, Am Handelshafen 12, 27570 Bremerhaven, Germany; 4 Institute for Chemistry and Biology of the Marine Environment (ICBM), School of Mathematics and Science, Carl von Ossietzky Universität Oldenburg, Ammerländer Heerstraße 114-118, 26129 Oldenburg, Germany

**Keywords:** Evolution & Ecology, History & Philosophy of Science

## Abstract

The moon has significant impact on the timing of organisms. Can the study of molecular timing mechanisms of marine animals and algae help to understand some of the “weird” correlations between human physiological/behavioral rhythms and the lunar cycle?

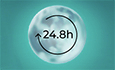

Luna alit ostrea et implet echinos (Lucilius, ca. 150BC)

## The moon and mythology

Since the dawn of mankind, the moon and its nocturnal cycle has held a special place in human myths and legends, often ascribing the Earth’s satellite special powers over fertility and reproduction. The above quote—“The moon nourishes the mussels and inflates the urchins”, which reflects a report on the observations of fishermen in Aristotle’s work “On the Parts of Animals”—shows that already more than four millennia ago, people saw a connection between the phases of the moon and the physiology of animals. This view has been generalized and exaggerated over time. Sixteenth-century philosopher Francis Bacon cited a contemporary belief that “the brains of rabbits, woodcocks, and calves are largest at the full moon” (Sylva Sylvarum, 1627, cent.IX, sect.892).

While there is no scientific evidence for such effects on brains, detailed biological observations starting in the early and mid-20th century have now clearly established a connection between the apparent size of specific marine invertebrates with different phases of the moon, 2000 years after Lucilius’ quote. It seems that human myths and legends initially built on valid observations, namely that reproductive processes, including gonad size of certain marine species depend on the lunar cycle. In species, where the gonads contribute to a large proportion of the body mass, such as some sea urchins and mussels, this effect is particularly prominent and will reflect itself in the size and body masses of these animals, as if the moon inflated them. Like archeologists, who can take leads from historical texts and stories to search for evidence, biologists now disentangle truth from mythology in order to understand the extent and the mechanisms of the moon’s impact on life.

“It seems that human myths and legends initially built on valid observations, namely that reproductive processes […] depend on the lunar cycle.”

## Timing systems

**Figure 1:**
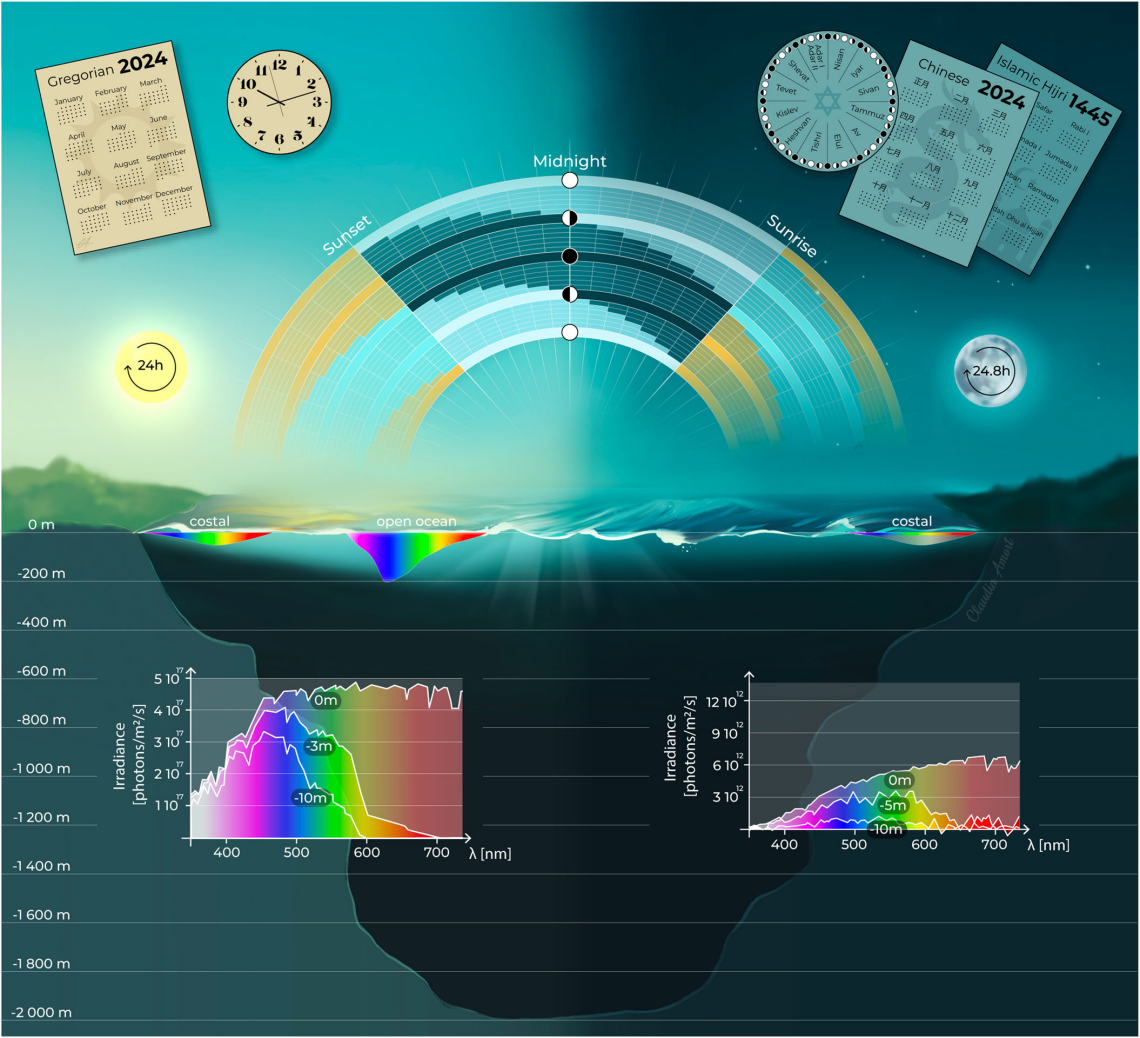
Sun and moon provide time information for human cultures and biological systems in their natural environment. Top: Left versus right represents existing solar (e.g., Gregorian) versus lunar (e.g., Chinese, Jewish, and Islamic) human calendar systems. Middle: Presence of the moon in the sky across the lunar phases relative to sunrise and sunset. Bottom: Representative solar versus lunar (full moon) illumination at the surface and different water depths. Spectra based on (Veedin Rajan et al, 2021; Zurl et al, 2022). Created by Claudia Amort under a CC-BY license.

Still, for many scientists, research to understand lunar effects on biology may appear dubious. However, it might be helpful to look at the timing systems used in various cultures to appreciate the value of celestial clocks. Many human cultures typically rely on the sun for setting the time of calendars and watches. Yet, by sheer numbers, probably most humans use lunar-based calendars, which are common in Chinese, Indian, Islamic, Jewish, and several indigenous native cultures. In terms of human cultural evolution, lunar-based calendars probably represent the earliest detectable evidence for timing systems, dating back to the Stone Age. Obviously, the moon provides reliable and trackable time information to organize human societies (Fig. 1).

“In terms of human cultural evolution, lunar-based calendars probably represent the earliest detectable evidence for timing systems, dating back to the Stone Age.”

Likewise, biological systems require temporal organization across different time periods. Research during the past 100 years has demonstrated the ubiquitous existence and importance of daily rhythms and timing systems, aka circadian clocks. These biological oscillators ensure that the organism keeps its time based on internal periodic processes. Biological oscillatory systems phase-synchronize by internal and/or external cues, but they do not depend on them. When properly functioning, circadian oscillators, which run with an intrinsic period length of about 24 h are—like any well-functioning biological process—subconscious. We only become aware of them when their normal functions get unhinged, such as during shift work or jet lags.

“The increasing knowledge about the molecular mechanisms underneath daily biological clocks led to an increased recognition of their widespread and fundamental importance.”

The increasing knowledge about the molecular mechanisms underneath daily biological clocks led to increased recognition of their widespread and fundamental importance from actin dynamics in liver cells (Gerber et al, 2013) and cell division in various tissues and species (reviewed in Brown, 2014) to complex processes like cancer (reviewed in Sulli et al, 2019), plant growth (reviewed in Greenwood and Locke, 2020) or animal behavior and cognition (reviewed in Häfker and Tessmar-Raible, 2020; Salehinejad et al, 2021). There is also little doubt that annual/seasonal timing is critical for many species (reviewed in Hafker et al, 2022; Häfker and Tessmar-Raible, 2020; Wirz-Justice, 2018). Both daily and annual timing is driven by the sun. But how about the impact of the lunar cycle on biological systems?

## Timing by the moon is phylogenetically and ecologically widespread

When it comes to understanding the potential effects of the moon on biology, it is obvious that organisms in tidal regions should be particularly prone to it since tidal cycles with a periodicity of about 12.4 h are caused by the gravitational influence of the moon (Kwiatkowski et al, 2024; Rock et al, 2022). The tides are obvious on the coast, but they even extend to the deep sea, where they cause significant and regular changes to local current flows (Mat et al, 2020) and to the land, as so-called solid earth tides (Lau and Schindelegger, 2023).

The tidal cycles themselves exhibit amplitude oscillations over a period of approximately 14.7 days. This is because superimposed on the lunar cycle are the 14.75 and 29.5 days periodicities when the sun and the moon are in linear relation to each other, thereby enhancing the gravitational forces.

However, like the sun, the moon also provides timing information besides gravitational changes—namely light intensity and spectrum—that can impact organisms. The most obvious periodicity is that of a full lunar cycle, which encompasses 29.5 days. For humans in urban areas, moonlight might not be such an obvious environmental stimulus, but those who experience truly dark nights without artificial light will notice how much difference the presence of moonlight can make. This is not just true on land, but also for the aquatic environment (Fig. 1).

“like the sun, the moon also provides timing information besides gravitational changes—namely light intensity and spectrum—that can impact on organisms.”

When considering biological timing by the moon, it is also important to note that tidal versus (semi)monthly periods are of quite different length and likely also have different underlying molecular mechanisms (Andreatta and Tessmar-Raible, 2020; Hafker et al, 2022). In other words, lunar timing is probably not all the same. To further complicate matters, accumulating evidence suggests that the underlying mechanisms will likely differ even within the same (semi)monthly period (Kaiser and Neumann, 2021), although at present, the data are too scarce on any moon-dependent timing mechanism to come to a well-founded conclusion.

## The role of the moon for humans and other animals

**Figure 2:**
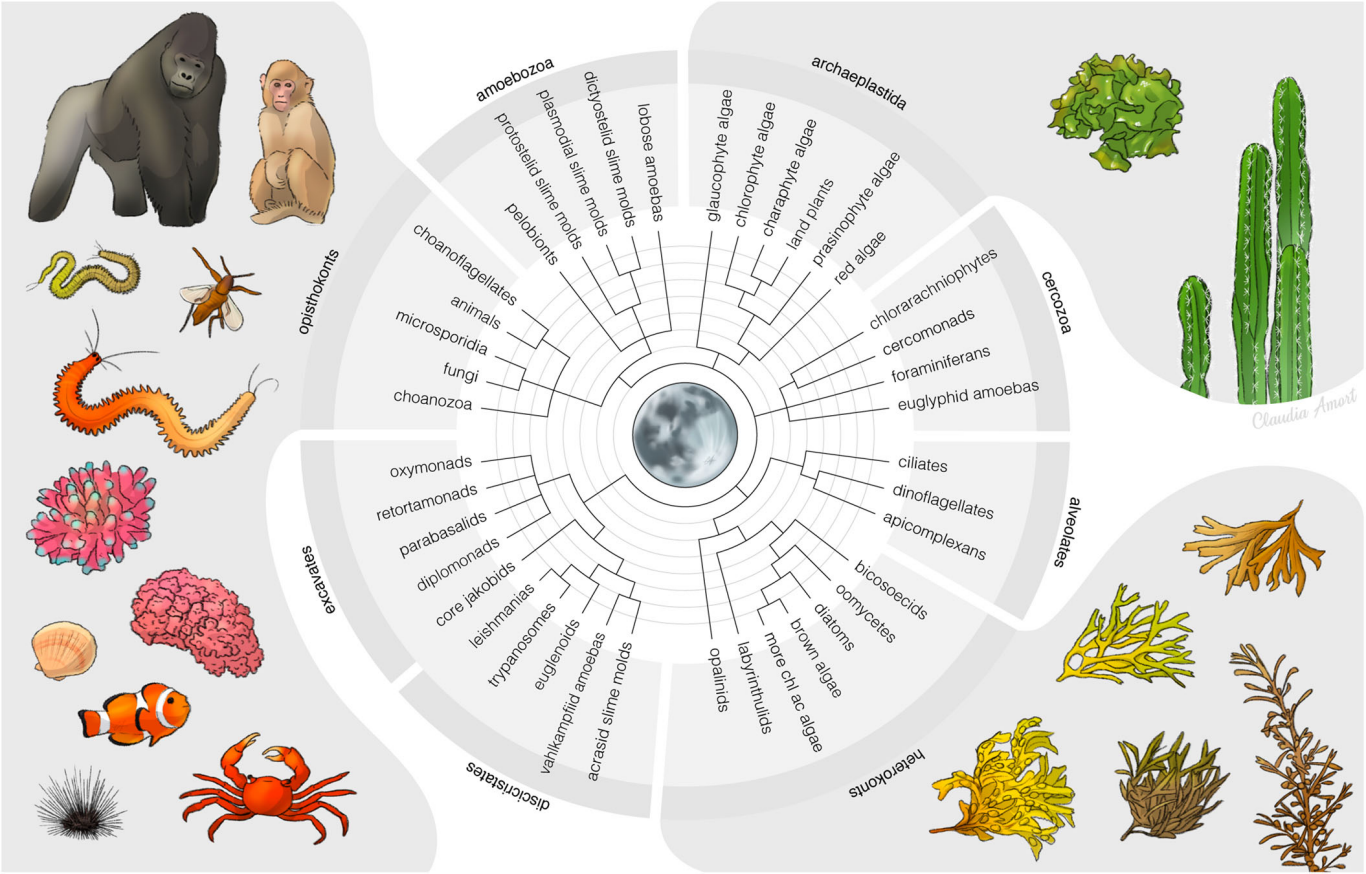
Moon-controlled monthly and semimonthly reproductive cycles are widespread across eukaryote phylogeny. Exemplary selection of species with moon-controlled reproductive cycles documented in the scientific literature. Phylogenetic tree based on (Baldauf, 2003). Created by Claudia Amort under a CC-BY license.

Monthly and semimonthly rhythms have been documented for a wide number of terrestrial and marine animal species (Fig. 2). In essence, there are two major and interlinked processes that are influenced by these rhythms: reproduction and daily activity/resting behavior. In many cases, the lunar rhythms are, in addition, modulated by annual cycles that, for example, result in monthly spawning events that only occur during a restricted time window, which can be as rare as once per year.

External fertilization is a frequent mode of reproduction in marine animals, and the lunar cycle provides a steady rhythm that can be used to synchronize spawning across a population and thus optimize reproductive success. Famous examples of such reproductive rhythms are the mass spawnings by corals, Samoan palolo worms or the great migration of the Christmas Island red crabs from the forests to the ocean to spawn (reviewed in Andreatta and Tessmar-Raible, 2020; Kaiser and Neumann, 2021). It is not clear yet whether these rhythms are triggered by the lunar light cycle or by the organisms’ internal clocks for most of these examples. Yet, a few marine species, in particular the midge *Clunio* and a close relative of the Samoan palolo, the bristle worm *Platynereis dumerilii*, have been extensively observed under lab conditions, which showed that they indeed possess inner oscillators of yet unknown molecular/cellular mechanisms (reviewed in Andreatta and Tessmar-Raible, 2020; Kaiser and Neumann, 2021). It is the phase of these oscillators that is entrained by full moonlight. While light is necessary and sufficient for phase synchronization of the *Platynereis* lunar oscillator (Zantke et al, 2013), it is likely that other cues, such as monthly tidal variations, will also be used under natural conditions. In fact, specific strains of *Clunio marinus* have lost their lunar light sensitivity and instead use the mechanical information provided by the tides (reviewed in Kaiser and Neumann, 2021).

The dependency on moonlight to synchronize reproduction can become a problem, in particular in the light of human civilization. Artificial light at night (ALAN) obviously disrupts light/dark cycles and can seriously threaten the reproductive rhythms of many marine organisms that use lunar light cycle to synchronize their reproduction or to entrain their inner oscillator (Jägerbrand and Spoelstra, 2023).

“The dependency on moonlight to synchronize reproduction can become a problem, in particular in the light of human civilization.”

More than 22% of coastal regions are exposed to anthropogenic light pollution (Marangoni et al, 2022), which has well-documented effects on biological rhythms, especially for corals (Ayalon et al, 2021). Analyses of long-time spawning records document a desynchronization for multiple species. Substantial evidence in *Acropora* species shows that ALAN disrupts natural moonlight cycles, which in turn impacts the dynamics of reproductive-tissue development (Ayalon et al, 2021).

These findings underscore the alarming implications of ALAN for coral reef ecosystems. Given that a significant proportion of marine organisms rely on synchronized spawning for optimizing reproduction, such disruptions likely compromise their reproductive success, which creates significant challenges for the resilience and recovery of already stressed coral reefs and many other marine ecosystems.

## The value of non-human model systems

It is clear that research into lunar-controlled biological rhythms and their mechanisms is not merely fascinating science, but critical to provide knowledge that will help to prevent or counteract the destructions caused by anthropogenic impacts, including ALAN. Understanding the features of the oscillatory systems are especially important given their limits of temperature compensation and ranges of entrainment (Häfker and Tessmar-Raible, 2020).

Moreover, there is increasing evidence that the menstrual cycle of great apes and humans is also influenced by the monthly lunar cycle (Fig. 2) (Graham, 1981; Helfrich-Forster et al, 2021). Menstrual disorders, including irregular cycles, oligomenorrhea and amenorrhea, occur in up to 75% of adolescent females, which makes them a significant public health concern (Odongo et al, 2023). Thus, a better understanding of human monthly oscillators and what could cause their disruptions can greatly benefit from a molecular mechanistic understanding of such oscillators in general.

As biological research has proven many times, model systems that can be functionally interrogated in the laboratory are critical to test for causal relationships, which will lead to molecular models and more generally applicable mechanisms. During the past years, four marine systems have been particularly promising to lift the fog on (semi)monthly lunar rhythms and clocks: the brown algae *Dictyota dichotoma* (Coelho and Cock, 2020), the midge *Clunio marinus* (Kaiser and Neumann, 2021), the annelid *Platynereis dumerilii* (Zantke et al, 2013) and the pufferfish *Takifugu alboplumbeus* (Chen et al, 2022). They cover very different phylogenetic positions, lifestyles, and habitats (Fig. 2), and thus will provide an overview of conserved and divergent principles of circalunar oscillators and their up- and downstream pathways.

## How do animals sense the lunar phases?

Like human calendars, the inner calendars of organisms need to be aligned, that is, phase-synchronized, to moon phase. Otherwise, the month would start on a different day for each individual. Indeed, the full moon is a necessary and sufficient cue for phase synchronization of the *Platynereis dumerilii’s* inner monthly oscillator. It raises the question of how these worms are able to determine the “right” moon phase? After all, moonlight is reflected sunlight, and what reaches the photoreceptor proteins are photons, irrespective of origin. A biological system that synchronizes to a specific moon phase thus needs to fulfill at least three conditions: the involved photoreceptors need a very high sensitivity and sensitivity range, given that moonlight is 10^5^–10^6^ orders of magnitude dimmer than sunlight (Fig. 1); the system needs to give the light a specific ‘ecological meaning’, that is, to discriminate if the spectral range and intensity correspond to moonlight or sunlight; and the system needs to decode the duration of moonlight.

Moon phases differ not just by the intensity of light, but by the duration of light during the night (Fig. 1). Thus, at a given geographic location, certain durations of moonlight will only be reached during the full moon and the days close to it. The exact durations of the full moon vary with the photoperiod, but the general rule that the full moon rises when the sun sets and sets when the sun rises is independent of photoperiod and geographic location (Hafker et al, 2022).

A possible mechanism how such a moon-phase detection system can work has recently been uncovered in *Platynereis dumerilii* (Fig. 2), which involves the L-Cry protein, a type 1 cryptochrome. In its dark state, it occurs as a dimer bound by correspondingly two molecules of FAD^+^ (Poehn et al, 2022). Oligomers might exist too, but at present, the dimer-state is best verified.

Biochemical, structural, genetic, and cell biological data suggest a model, in which this cryptochrome has different quantum efficiencies for the photoreduction reactions of its two FAD^+^. While the first reaction has a high efficiency that allows the low-intensity moonlight to photoreduce the first FAD+, the second photoreduction requires an amount of energy that can only be achieved by sunlight. The hypothesis is that photoreduction of the first FAD+ changes the dimer conformation, which reduces the quantum efficiency for the second reaction (Poehn et al, 2022). Experimental data also suggest that L-Cry can photo-accumulate. This would imply that the amount of L-Cry dimers with one reduced FAD and one oxidized FAD+ (“moonlight state”) is proportional to the duration of moonlight (Fig. 1).

In the test tube, purified L-Cry takes 6 h and more to be fully converted to its moon-light state, a duration that, at the natural habitat of the worms, is only reliably reached around the full moon (Hafker et al, 2022; Poehn et al, 2022), Fig. 1). The cellular localization of the different L-Cry dimer states differ correspondingly, suggesting differential signaling pathways (Poehn et al, 2022; Zurl et al, 2022). Of note, *l-cry* mutant worms are still able to sense and entrain moonlight, but they lose the ability to discriminate between the light they should entrain to versus ecologically “wrong” light. Thus, L-Cry likely functions as a “light interpreter” (Poehn et al, 2022) by regulating the information flow of other, yet unknown, photoreceptor(s) to the oscillator.

A commonly asked question is how moonlight-dependent systems work during clouded nights or turbid waters. It is important to remember that lunar-period depending organisms entrain their oscillatory systems. This means that these oscillators, once synchronized, can run independently of moon or any other light over several months (Kaiser and Neumann, 2021; Zantke et al, 2013). Thus, any rhythmic output will continue irrespective of clouds or turbid waters.

## The human condition

Monthly oscillators also synchronize the reproductive circalunar cycles of human females (Helfrich-Forster et al, 2021), other great apes such as orang utans and gorillas (Graham, 1981) (Fig. 1). The question remains, especially for humans, whether they are synchronized by the lunar environmental cycle, for which there is some evidence (Helfrich-Forster et al, 2021). What seems to cause this uncertainty is the fact that the phase of the human female circalunar oscillator and the environmental lunar cycle are not always aligned.

Is this variance due to low numbers of study individuals and, hence, statistical artifacts? Or is it caused by differences in local environmental parameters, either of natural or anthropogenic origin? Could there be different susceptibilities depending on genetic background and/or age? A combination of all these factors?

What is clear is that (semi)monthly reproductive cycles synchronized by the moon are very widespread in the animal kingdom (Fig. 2), and humans are just one type of animal. After all, it might not be too surprising if lunar effects were still observable in our biology. Why we and other terrestrial animals have such (semi)monthly cycles is still largely unclear. It might simply be an advantage to not leave biological processes up to temporal randomness and to employ timers at different period lengths, like watches and calendars are useful for the organization of human societies.

“What is clear is that (semi)monthly reproductive cycles synchronized by the moon are very widespread in the animal kingdom […], and humans are just one type of animal.”

## The modulation of daily timing by the lunar cycle

A second aspect at which the monthly lunar cycle exerts influences on biology is the modulation of daily activity/resting behaviors. By way of example, the annelid *Platynereis dumerilii* adjusts its nocturnal swarming hour depending on the phase of the month. This is likely achieved by free-running – that is, no phase resetting by sunlight—a plastic circadian-circalunidian clock with a period length of ~24.8 h during the waning quarter moon, followed by normal entrainment to sunlight until the subsequent full moon nights. The entrainment to sunlight results in a constant spawning time every 24 h at the end of the night. Finally, in order to reach the early darkness hours of the waning moon (Fig. 1), the plastic circadian-circalunidian clock exhibits a <24 h period length under full moonlight (Zurl et al, 2022).

Of note, the resulting behavioral modulations are reminiscent of changes seen in human sleep patterns across the month/lunar cycle (e.g., Cajochen et al, 2013; Casiraghi et al, 2021). However, these findings in humans have remained controversial given that different studies keep producing different results. Yet, it should be considered that human studies are more difficult to control than lab animal studies. The current status might be best summarized by quoting a recent overview article: “Longitudinal observations call into question the scientific consensus that humans are unaffected by lunar cycles” (Wehr and Helferich-Forster, 2021). Hence, non-human model systems will need to pave the way for a deeper understanding of possible molecular and cellular mechanisms that underly the influence of the moon even on humans.

## The role of moon outside the animal kingdom

While the lunar phases affect various aspects of animal biology, its influence extends to terrestrial plants and seaweeds, which reveals a fascinating interplay between flora and the lunar cycle (Fig. 2). One striking example of lunar synchronization is the blossoming of *Cereus peruvianus*, commonly known as the Peruvian apple cactus. This majestic columnar cactus originates from South America, where it blossoms during summer nights. Furthermore, its flowering pattern oscillates predictably in synchrony with the lunar phases, resulting in waves that overlap with the full moon’s glow. But what drives this floral synchrony during full moon nights? The answer to this question could lie in the partnership between the cactus and a nocturnal bat pollinator. As dusk descends and the moon rises, the cactus adorns itself with large, cream-colored flowers. These ephemeral flowers open for just a single night, likely serving as a crucial nectar source for the bats.

In the shadowy world of the moonlight, another intricate dance unfolds between night-flying insects and the plant *Ephedra foeminea* (Rydin and Bolinder, 2015). Pollination of this species is orchestrated by night-flying dipterans and lepidopterans, which are attracted by nectar droplets produced during the full moon phase. A proposed hypothesis suggests that these droplets act as mirrors that reflect the moonlight and thereby visually attract pollinators to the flowers.

“In the shadowy world of the moonlight, another intricate dance unfolds between night-flying insects and the plant *Ephedra foeminea*.”

Besides terrestrial plants, the timing dictated by lunar cycles holds significant influence over the biology of various seaweed species, encompassing green (*Chlorophyta*) and brown algae (*Phaeophyceae*) from disparate evolutionary lineages (Fig. 2). These sessile macroorganisms share habitats and seem to have thus evolved convergent adaptive strategies, particularly in response to environmental factors such as tides. These green and brown algae undergo sexual reproduction via external fertilization in the water column, necessitating precise timing for gamete mating (Pearson and Serrão, 2006). Long-standing observations showed the occurrence of spawning in semilunar rhythms, where reproductive activities coincide with particular tidal conditions, often during full and new moons, that is, during spring tides (Hoyt, 1907; Monteiro et al, 2016).

Like for animals, the fertilization success of plants largely depends on the frequency of gamete encounters. By aligning spawning with lunar cycles, there is an increased likelihood of gamete encounters, thereby enhancing the species’ fitness. For example, studies on several brown algal species within the genus *Fucus* showed that spawning and embryo settlement happens on a daily and semilunar fashion during neap tides (Pearson and Serrão, 2006, Monteiro et al, 2016).

The precise coordination in reproductive timing is also synchronized such that it plays a crucial role in maintaining species boundaries, particularly among closely related species that coexist in the same habitat. Indeed, maintaining reproductive isolation in marine species that operate through external fertilization are challenging, and temporal differences in gamete release, therefore, prevent cross-reproduction (Palumbi, 1994). Hybrids often exhibit lower fitness than their parental lineages, and for this reason there is a strong selection pressure for setting asynchronous timing of reproduction in species that could potentially hybridize. Several sexually compatible *Fucus* share the same habitat along the intertidal zone. They all spawn within the same semilunar pattern, but at different times of the day. This divergence in timing creates distinct temporal niches, contributing to prevent cross-reproduction (Monteiro et al, 2016).

## How do algae sense the lunar phases?

Again, as in animals, synchronizing to lunar cycles involves internal biological clocks entrained by environmental cues. Despite the limited understanding of the lunar-timed clocks in brown algae, it appears that they integrate several information sources to establish semilunar rhythms (Fig. 1). Coastal areas under tidal influence are unique oscillatory environments where the day-night cycle ads to the regular variations in water depth induced by the tides. The seawater selectively absorbs visible wavelengths as depth increases (Fig. 1), and for this reason, the water column acts as a light filter, altering light spectrum and intensity. These periodic light variations provide crucial timing information about the lunar and tidal cycles.

Marine algae are equipped with photoreceptors capable of detecting changes in their photic environment. While certain photoreceptors are present in all algae, others are specific to particular lineages (Jaubert et al, 2017; Mat et al, 2024). Although the photoreceptors responsible for moonlight perception in seaweeds remain unidentified, the illumination during full moon acts as a vital signal for coordinating semilunar rhythms in brown algae, implying their role in synchronizing the circalunar clock (Kaiser and Neumann, 2021)

Early work on the brown algae *Dictyota dichotoma* demonstrated the presence of an endogenous circalunar clock (Kaiser and Neumann, 2021). Experimental manipulation through regular artificial moonlight cycles showed semilunar spawning rhythms lasting for approximately 14 days. Notably, these rhythms can persist for weeks even in the absence of lunar cues, indicating independent timekeeping mechanisms. Attempts to decipher the underlying mechanisms of this clock have led to the formulation of a ‘beat’ coincidence model (Kaiser and Neumann, 2021). It suggests that semilunar rhythms result from the overlapping oscillations of both a circadian and a circatidal clock, aligning every two weeks to generate semilunar beats. Additionally, this mechanism is proposed to be synchronized on a monthly basis by the illumination of full moon nights. However, the precise manner in which moonlight synchronizes fortnightly rhythms of seaweeds, as well as the specific components of the circatidal and circadian clocks, remains elusive even after six decades of work. These lines of study present intriguing avenues for exploration by chronobiologists.

After decades of research into biological clocks, their entrainment by environmental cues, and their effects on organisms’ behavior and reproduction, it seems that our ancestors were, after all, quite right by associating the moon with romance and fertility.

BOX: Further reading **The moon as a cultural timing system**

Boyle R, 2019. Ancient humans used the moon as a calendar in the sky, ScienceNews. Society for Science: EIN 53-0196483, https://www.sciencenews.org/article/moon-time-calendar-ancient-human-art.

Marshack A (1971) *The Roots of Civilization: The Cognitive Beginnings of Man’s First Art, Symbol, and Notation*. McGraw-Hill

Rappenglück MA (2015) Possible Astronomical Depictions in Franco-Cantabrian Paleolithic Rock Art. In: *Handbook of Archaeoastronomy and Ethnoastronomy*, Ruggles C.L.N. (ed.) pp. 1205-1212. Springer New York: New York, NY

**Lunar timing in marine organisms**

Andreatta G, Raible F, Tessmar-Raible K (2022) Biological rhythms: hormones under moon control. Curr Biol 32: R1269-R1271

Berndt ML, Callow JA, Brawley SH (2002) Gamete concentrations and timing and success of fertilization in a rocky shore seaweed. Marine Ecol Prog Ser 226: 273-285

Bogaert K, Beeckman T, De Clerck O (2016) Abiotic regulation of growth and fertility in the sporophyte of Dictyota dichotoma (Hudson) J.V. Lamouroux (Dictyotales, Phaeophyceae). J Appl Phycol 28: 2915–2924

Briševac D, Prakash C, Kaiser TS (2023) Genetic analysis of a phenotypic loss in the mechanosensory entrainment of a circalunar clock. PLoS Genet 19: e1010763

Kaiser TS, Poehn B, Szkiba D, Preussner M, Sedlazeck F, Zrim A, Neumann T, Nguyen L-T, Betancourt AJ, Hummel T et al (2016) The genomic basis of circadian and circalunar timing adaptations in a midge. Nature 540: 63–73

Kumke J (1973) Beiträge zur periodizität der Oogon-Entleerung bei *Dictyota dichotoma* (Phaeophyta). Zeitschrift für Pflanzenphysiologie 70: 191–210

Levitan DR, Fogarty ND, Jara J, Lotterhos KE, Knowlton N (2011) Genetic, spatial, and temporal components of precise spawning synchrony in reef building corals of the Montastraea annularis species complex. Evolution 65: 1254–1270

Lüning K, Kadel P, Pang S (2008) Control of reproduction rhythmicity by environmental and endogenous signals in *Ulva pseudicurvata* (chlorophyta). J Phycol 44: 866–873

Neumann J, Rajendra D, Kaiser TS (2024) The free-running circasemilunar period is determined by counting circadian clock cycles in the marine Midge Clunio Marinus. J Biol Rhythms 7487304241249516

Özpolat BD, Randel N, Williams EA, Bezares-Calderón LA, Andreatta G, Balavoine G, Bertucci PY, Ferrier DEK, Gambi MC, Gazave E et al (2021) The Nereid on the rise: Platynereis as a model system. Evodevo 12: 10

Raible F, Takekata H, Tessmar-Raible K (2017) An overview of monthly rhythms and clocks. Front Neurol 8:189

Smith GM (1947) On the reproduction of some pacific coast species of *Ulva*. Am J Bot 34: 80–87

Tessmar-Raible K, Raible F, Arboleda E (2011) Another place, another timer: marine species and the rhythms of life. Bioessays 33: 165–172

Vu HH, Behrmann H, Hanic M, Jeyasankar G, Krishnan S, Dannecker D, Hammer C, Gunkel M, Solov’yov IA, Wolf E et al (2023) A marine cryptochrome with an inverse photo-oligomerization mechanism. Nat Commun 14: 6918

Wichard T, Charrier B, Mineur F, Bothwell JH, Clerck OD, Coates JC (2015) The green seaweed Ulva: a model system to study morphogenesis. Front Plant Sci 6:72

**Lunar effects on land plants**

Ben-Attia M, Reinberg A, Smolensky MH, Gadacha W, Khedaier A, Sani M, Touitou Y, Boughamni NG (2016) Blooming rhythms of cactus Cereus peruvianus with nocturnal peak at full moon during seasons of prolonged daytime photoperiod. Chronobiol Int 33: 419–430

**Anthropogeneic effects**

Davies TW, Levy O, Tidau S, de Barros Marangoni LF, Wiedenmann J, D’Angelo C, Smyth T (2023) Global disruption of coral broadcast spawning associated with artificial light at night. Nat Commun 14: 2511

Shlesinger T, Loya Y (2019) Breakdown in spawning synchrony: a silent threat to coral persistence. Science 365: 1002–1007

The possible influence of the moon on monkey, great ape and human biology

Ecochard R, Stanford JB, Fehring RJ, Schneider M, Najmabadi S, Gronfier C (2024) Evidence that the woman’s ovarian cycle is driven by an internal circamonthly timing system. Sci Adv 10: eadg9646

Habumuremyi S, Stephens C, Fawcett KA, Deschner T, Robbins MM (2016) Endocrine assessment of ovarian cycle activity in wild female mountain gorillas (Gorilla beringei beringei). Physiol Behav 157: 185–195

Walker ML, Wilson ME, Gordon TP (1984) Endocrine control of the seasonal occurrence of ovulation in rhesus monkeys housed outdoors. Endocrinology 114: 1074–1081

Wehr TA (2018) Bipolar mood cycles and lunar tidal cycles. Mol Psychiatry 23: 923–931

Weinbauer GF, Niehoff M, Niehaus M, Srivastav S, Fuchs A, Van Esch E, Cline JM (2008) Physiology and endocrinology of the ovarian cycle in macaques. Toxicol Pathol 36: 7s–23s

### Supplementary information

Peer Review File
