# Supplementary material for: Time me by the moon: The evolution and function of lunar timing systems
Source: EMBO Rep. 2024 Jul 16;25(8):3169–76. doi: 10.1038/s44319-024-00196-5 (PMC11316100; doi:10.1038/s44319-024-00196-5)
Supplement: Supplementary file 1 — Peer Review File [file 44319_2024_196_MOESM1_ESM.pdf]

EMBO reports

Time me by the moon

Kristin Tessmar-Raible and Andrés Ritter

Corresponding author(s): Kristin Tessmar-Raible (kristin.tessmar@mfpl.ac.at) , Andrés Ritter (aritter@sb-roscoff.fr)

Review Timeline:

Submission Date:	19th Jun 24
Accepted:	24th Jun 24

Editor: Holger Breithaupt

Transaction Report: This article was editorially reviewed.
